# Predictive validity of the reduced Cariogram model for caries increment in non-cavitated and cavitated lesions: cohort study

**DOI:** 10.1186/s12903-023-03479-w

**Published:** 2023-10-24

**Authors:** Muhammad Taqi, Syed Jaffar Abbas Zaidi

**Affiliations:** 1 Department of Community Dentistry Dow Dental College, Dow University of Health Sciences, Karachi, 74200 Pakistan; 2 Department of Oral Biology, Dow Dental College, Dow University of Health Sciences, Karachi, Pakistan

**Keywords:** Cariogram, ICDAS, Dental caries, Childhood caries, Caries prevention

## Abstract

**Background:**

The aim of this study is to assess the caries prediction of the reduced Cariogram by comparing baseline caries risk profiles with non-cavitated and cavitated lesions over periods of six, twelve, and 18 months.

**Methods:**

From May 2016 to October 2017, seven schools in Bhakkar, Pakistan, participated in a cohort study. First base line examination was conducted followed by examinations at 6, 12 and 18 months. Children intraoral examinations were performed on portable dental chair with in school premises by a trained examiner. A modified ICDAS index was used to measure caries at baseline and at follow-up examinations after 6, 12, and 18-months. A receiver operating curve (ROC) analysis was performed to evaluate its effectiveness for predicting dental caries increment.

**Results:**

About 40% of children had a low-risk status, 30.5% medium risk, and 29.7% high risk, at baseline risk assessment. At 18 months, 73% of high-risk children, 59% of medium-risk children, and 41% of low-risk children showed a caries increment. For the reduced Cariogram model, the area under the curve on the 6, 12 and 18 months follow-up was 0.63, 0.65 and 0.70 respectively.

**Conclusions:**

Our findings indicates that a reduced Cariogram can predict the progression of caries in both cavitated and non-cavitated lesions and model exhibits a level of discriminatory ability. While it might not achieve a very high accuracy, it suggests that the model is able to predict caries increment effectively than random guessing.

## Introduction

The contemporary approach to caries management involves risk-based, patient-centered strategies [1]. The effectiveness of preventive measures depends on the availability of an accurate caries risk assessment system that can determine an individual's level of caries risk [1]. To address the complexity of caries risk, a computerized program called Cariogram has been developed as a valuable tool for evaluating caries risk [2].

Cariogram was specifically developed to establish the connection between caries and its risk factors. It was designed to provide insights into the likelihood of caries occurrence, present a graphical representation of caries risk, and recommend appropriate preventive treatments [3]. There are two versions of Cariogram available: the full version and the reduced version. The full Cariogram employs nine caries-related factors to establish a comprehensive caries risk profile, serving as both a predictor and risk model to assist in intervention planning [4]. On the other hand, the reduced Cariogram utilizes seven caries-related factors to establish a simplified caries risk profile [4].

Numerous studies have explored the predictive validity of the reduced Cariogram in assessing dental caries increment among children aged 12 and preschoolers over a 12–24-month period, with cavitated lesions serving as indicators [5, 6]. However, there is a noticeable gap in the literature examining the predictive value of the reduced Cariogram in identifying non-cavitated carious lesions as early as six months. It is crucial to recognize that excluding non-cavitated lesions from this analysis could result in a significant loss of valuable information since these lesions contribute substantially to caries experience [7]. Furthermore, dental treatment planning often primarily targets cavitated lesions, thereby overlooking the importance of non-cavitated lesions.

In countries like Pakistan, where there is a high prevalence of caries and limited resources for oral healthcare services, the ability of the reduced Cariogram to predict the early onset of non-cavitated lesions can aid clinicians in managing treatment intensity. This also enables efficient allocation of treatment resources to those genuinely in need [7]. Therefore, the main objective of our study was to assess the predictive ability of the reduced Cariogram for caries by comparing baseline caries risk profiles with non-cavitated and cavitated lesions over periods of six, twelve, and eighteen months.

## Methods

From May 2016 to October 2017, seven schools in Bhakkar, Pakistan, participated in a cohort study. First base line examination was conducted followed by examinations at 6, 12 and 18 months. The Medical Ethics Committee of the Faculty of Dentistry at the University of Malaya approved this study (Ref no. DF 71 CO1512/0072(P)). This study was permitted to be conducted in government schools by the district education officer. Separate consents were sought from the administrations of all private schools.

Parents' written consent was obtained before children were selected. The study participants were conveniently selected. Children aged 11–12 who understood basic instructions were conveniently included in this study. Children who are younger than or older than 11–12 years of age, receiving orthodontic treatment, and with acquired physical disabilities were excluded from the study. Children who required urgent care during the examination were referred to the nearest district hospital for dental treatment.

We calculated the sample size using Lawrence & Sheiham's [8] mean caries progression rates. The following formula was used to calculate the sample size *n* = (Zα/2 + Zβ / ES)^2^ [9]. Where ES = 0.36, Zα/2 = 1.96, Zβ = 0.84 (80%).

ES is effect size, Zα and Zβ are critical values from the standard normal distribution, corresponding to the chosen significance level (α) and the desired statistical power (1 − β) respectively.

Estimated sample size was 60 participants. Therefore, sixty participants were selected for each risk group. A sample size of 180 participants was estimated. Based on an attrition rate of 20%, a sample size of 226 was required.

### Risk assessment

At baseline, Cariogram was used to determine the risk of caries among the subjects based on seven factors. The DMFT index and Silness and Loe index were used to collect clinical observations related to "caries experience" and "plaque content". Parent-proxy questionnaires were used to collect information on caries-related systemic diseases and fluoride exposure. The decision regarding "clinical judgment" was reached by considering the combined scores of specific factors obtained from the Cariogram, along with sociodemographic factors. Through the use of a diet diary, we estimated the frequency of meals and snacks, as well as the contents of the diet. To verify the accuracy of the diet diary information, the subjects were interviewed after the diet diary was submitted. The score range for each factor is from 0–3 and 0–2 in case of systemic diseases [3].

The explanations of scorings for each factor according to Cariogram are shown below.

1. For the caries experience, examiner judgement can be used to choose the right score on the basis of a previous epidemiological survey [3]. For caries experience, the scores range from 0–3.
  Code 0: DMFT score 0
  Code 1: DMFT score of 1 and 2
  Code 2: DMFT score of 3 and 4
  Code 3: DMFT score of 5 and 6 or more
2. Plaque levels were measured using Loe and Sillness plaque index. The index teeth used to examine plaque levels were 16, 12, 24, 36, 32, and 44. For each index tooth, plaque score was estimated by adding the scores of the tooth surfaces (buccal, lingual, mesial and distal) and divided them by 4. Similarly, for an individual, the plaque score calculated for the six index tooth were added and divided by the total number of index teeth.
  Code 0: Extremely Good, Plaque index < 0.4.
  Code 1: Good, Plaque index 0.4–1.0.
  Code 2: Less than good, Plaque index 1.1–2.0.
  Code 3: Poor, Plaque index > 2.0.
3. The clinical judgement (perceptions of the examiner) factor is different from the other elements. It provides an opportunity for the examiner to express his/her ‘Clinical feeling’ and to see if the opinion differs from the program's inbuilt estimation. The default setting Code 1 was overruled by the examiner if not satisfied with the scoring [3].
  Code 0: The total impression of the caries situation, including social factors, gives a favourable view, more positive than what the Cariogram seems to indicate.
  Code 1: The Cariogram shows the risk, according to the other values entered.
  Code 2: If caries situation is worse than what Cariogram shows and the combined effect of dental caries including social factors, points in the direction of increased caries risk. Less than good compared to what the tests and the other factors seem to indicate.
  Code 3: The combined effect of the caries situation, including social factors, is very bad and the investigator is sure that caries will occur in the coming year and would want the green sector to be minimal.
4. Diet content and diet frequency analysis was based on the information given in the 3- day diet diary. Codes used for this factor are given below:
  Code 0: Very low intake of fermentable carbohydrates.
  Code 1: Low intake of fermentable carbohydrates/ non-cariogenic diet.
  Code 2: Moderate intake of fermentable carbohydrates.
  Code 3: Highly intake of fermentable carbohydrates/ high cariogenic diet.
5. For diet frequency, the Cariogram provides codes 0–3.
  Code 0: Maximum three sugar containing meals per day (including snacks).
  Code 1: Maximum five sugar containing meals per day.
  Code 2: Maximum seven sugar containing meals per day.
  Code 3: More than seven sugar containing meals per day.
6. The information related to fluoride exposure was gathered by interviewing the participants. For fluoride program, Cariogram provides Codes ranging from 0–3.
  Code 0: Maximum fluoride exposure based on frequent use of fluoride toothpaste, fluoride varnish, rinsing and tablets.
  Code 1: Infrequent use of fluoride toothpaste, fluoride varnish, rinsing and tablets.
  Code 2: Use of fluoride toothpaste only. Code 3: Not using any fluoride measures.
7. The factor of the general systemic disorder factor has three codes:
  Code 0: Healthy patients without any caries-related disease.
  Code 1: Patient with a disease which can indirectly contribute towards high caries risk.
  Code 2 was scored for bedridden patients or taking medication which can affect the saliva secretion.

After all information was collected and entered the Cariogram, each child was classified into a low, moderate, or high-risk group for caries. The Cariogram estimate the caries risk by using a complicated formula containing many ‘if’ conditions. All factors have been given a specific weight according to the chosen score, and as the scores increase the weight of that factor will be higher.

For example, in the default settings, the frequency of food intake factor has a higher weight than factors related to diet content. Similarly, the factor of bacterial counts has a higher weight as compared to the factor of plaque measurements. If two factors in the same group, such as diet content and frequency of food intake have high scores, additional weight to risk is given. It is also applicable for plaque amount and mutans streptococci counts. Addition weight is added to risk if several groups have high scores.

In case of non-use of fluoride heavyweight to the risk will be added. Cariogram also considers the operator’s ‘clinical judgment’, and weights to the risk are added depending on the score selected for an individual [10].

The weights associated with factors in Cariogram are created by the interpretation of data from numerous epidemiological, clinical studies and case reports from the literature in which various factors have been compared to caries incidence. The program contains about 5 million combinations of factors, and how the outcome for each combination will be can only be seen in the program [10].

After a caries risk assessment, all participants were invited to participate and allocated to the strata formed according to the risk level of participants. The baseline examination was then performed, followed by follow-up examinations with a gap of six months between each assessment. Before intra-oral assessment, a list of enrolled students containing only their name and class was provided to the school administration. On the day of the examination, a teacher nominated by the school administration called students from their classes according to the list provided.

### Calibrations

A single examiner was trained in ICDAS and calibrated. ICDAS Task Force member from Faculty of Dentistry, University Malaya, provided training about the modified ICDAS index coding procedure. After the training exercise, a calibration procedure was conducted a week later. The calibration process was conducted using the ICDAS codes for the 62 mounted teeth that were validated by the aforementioned task group. Cohen's weighted kappa was utilized to assess both intra- and inter-examiner reliability, yielding substantial agreement with an inter-examiner kappa value of 0.69 and an intra-examiner kappa value of 0.82 [11].

### Instrument & Intra oral examination

A modified ICDAS index was used to measure non cavitated and cavitated caries at baseline and at follow-up examinations after 6, 12 and 18 months. The modified ICDAS combined codes 1 and 2 and named them code A since compressed air could not be used to dry tooth surfaces [12]. However, codes 3–6 are cavitated carious lesions. In school premises, intraoral exams were conducted on portable dental chair.

### Dental caries increment estimation

The Adjusted Caries Increment (ADJCI) was used to quantify the rate of dental caries increment between cavitated and non-cavitated lesions [13].

$$\mathrm{ADJCI}\:=\:\left(\mathrm{Progression}\:\times\:\mathrm{Noprogressionnor}-\mathrm{regression}\right)/\left(\mathrm{Regression}\:+\:\mathrm{Noprogressionnor}-\mathrm{regression}\right).$$

### Statistical analysis

Descriptive analysis was used to calculate response rates, children who attended follow-ups, and dental caries increment. The level of significance was set at less than 0.05. Using the chi-square test, we measured the association between sociodemographic factors and caries risk levels.

Data of complete cases, absentee and drop out was entered in the Stata software. The characteristics of participants missing and those who completed the study was compared using chi-square test. Study variables caries risk level, and baseline modified ICDAS scores were used as predictors for multiple imputations. To evaluate the assumption that data were missing at random, the difference between individuals with complete and incomplete data were measured using Chi-square analysis. The chained equation was applied to impute missing values by the predictive regression model.

We employed a standard approach to dichotomize the continuous variable in order to assess the diagnostic accuracy of the tested tool. Specifically, we utilized Youden's J statistic to determine the optimal threshold for dichotomization.

Youden's index was applied for selecting the threshold that maximizes the sum of sensitivity and specificity, effectively balancing the trade-off between true positives and true negatives.

Once the optimal threshold was identified, data points above this threshold were categorized as "positive," while those below were categorized as "negative." This dichotomization enabled us to proceed with constructing the ROC curve, calculating sensitivity and specificity, and assessing the overall diagnostic performance of the tested tool.

## Results

From the list of names provided by schools, 400 children were initially invited. Out of which 300 submit signed parental consent, 70 students refused to take part in study and four students left school. Therefore, the final sample size was 226. From each school on average school 57 students were enrolled.

Table 1 presents the dropout and response rates of participants. Missing data accounted for 36.2% of this study, reducing sample size power to 70%. The multiple imputation method was used to generate plausible data for missing participants.

**Table 1:** Dropout and response rates of participants

	Risk assessment	Baseline	1^st^ Follow-up	2^nd^ Follow-up	3^rd^ Follow-up
Attendees	226	220	174	174	183
Absentees	0	6	22	11	0
Drop out	0	0	30	41	43
Total response rate	100%	97%	77%	77%	81%

Table 2 demonstrates that there are no statistically significant differences between participants who completed the study and those who dropped out across different caries risk categories and follow-up periods.

**Table 2:** Comparing the participants key exposure and ADJCI with complete and incomplete data

Risk category	Follow-up 1		*p*-value	Follow-up 2		*p*-value	Follow-up 3		*p*-value
	Observed	Missing		Observed	Missing		Observed	Missing	
Low	75 (83.3)	15 (16.7)	0.11	75 (83.3)	15 (16.7)	0.12	77 (84.4)	13 (15.6)	0.35
Medium	48 (69.4)	21 (30.4)		48 (69.6)	21 (30.4)		53 (75.4)	16 (24.6)	
High	51 (76.1)	16 (28.4)		51 (76.1)	16 (23.9)		53 (79.1)	14 (20.9)	

In Table 3, sociodemographic characteristics are compared to caries risk groups as determined by the reduced Cariogram. About 40% of children had a low-risk status, 30.5% medium risk, and 29.7% high risk, at baseline risk assessment. A significantly higher proportion of low-risk participants attended private schools (76.6%) than government-funded schools (20.8%) (*p* = 0.001).

**Table 3:** Comparison of sociodemographic variables with risk categories

Sociodemographic background	Caries risk levels according to Cariogram, n(%)			*p*- value
	Low	Moderate	High	
All subjects	90 (39.8)	69 (30.5)	67 (29.7)	
Male	49 (39.8)	38(30.9)	36(29.3)	0.98
Female	41(39.8)	31(30.1)	31(30.1)	
Urban	77(41.4)	53(28.5)	56(30.1)	0.34
Rural	13(32.5)	16(40)	11(27.5)	
Public funded	31(20.8)	63(42.3)	55(36.9)	.0001*
Private funded	59(76.6)	6(7.8)	12(15.6)	

Chi-square test

*Significant *p*-value < 0.05

At 18 months, 73% of high-risk children, 59% of medium-risk children, and 41% of low-risk children showed a caries increment. The occurrence of both cavitated and non-cavitated lesions was 74% at the initial assessment, 84% at the first subsequent evaluation, 81% at the second follow-up, and 83% at the third follow-up. Mean caries increment was consistently higher in participants with higher caries risk than low and moderate risk participants, as shown in Table 4.

**Table 4:** Mean adjusted caries increment (ADJCI) within each risk category at 6, 12 and 18 months

Risk category	Follow-up 1 (6 months) Mean ± SD	Follow-up 2 (12 months) Mean ± SD	Follow-up 3 (18 months) Mean ± SD
Low	0.84 ± 1.92	1.21 ± 3.38	1.11 ± 3.33
Medium	2.49 ± 3.29	2.41 ± 3.30	2.48 ± 4.11
High	3.01 ± 3.54	3.47 ± 3.82	4.01 ± 4.31

**Fig. 1:**
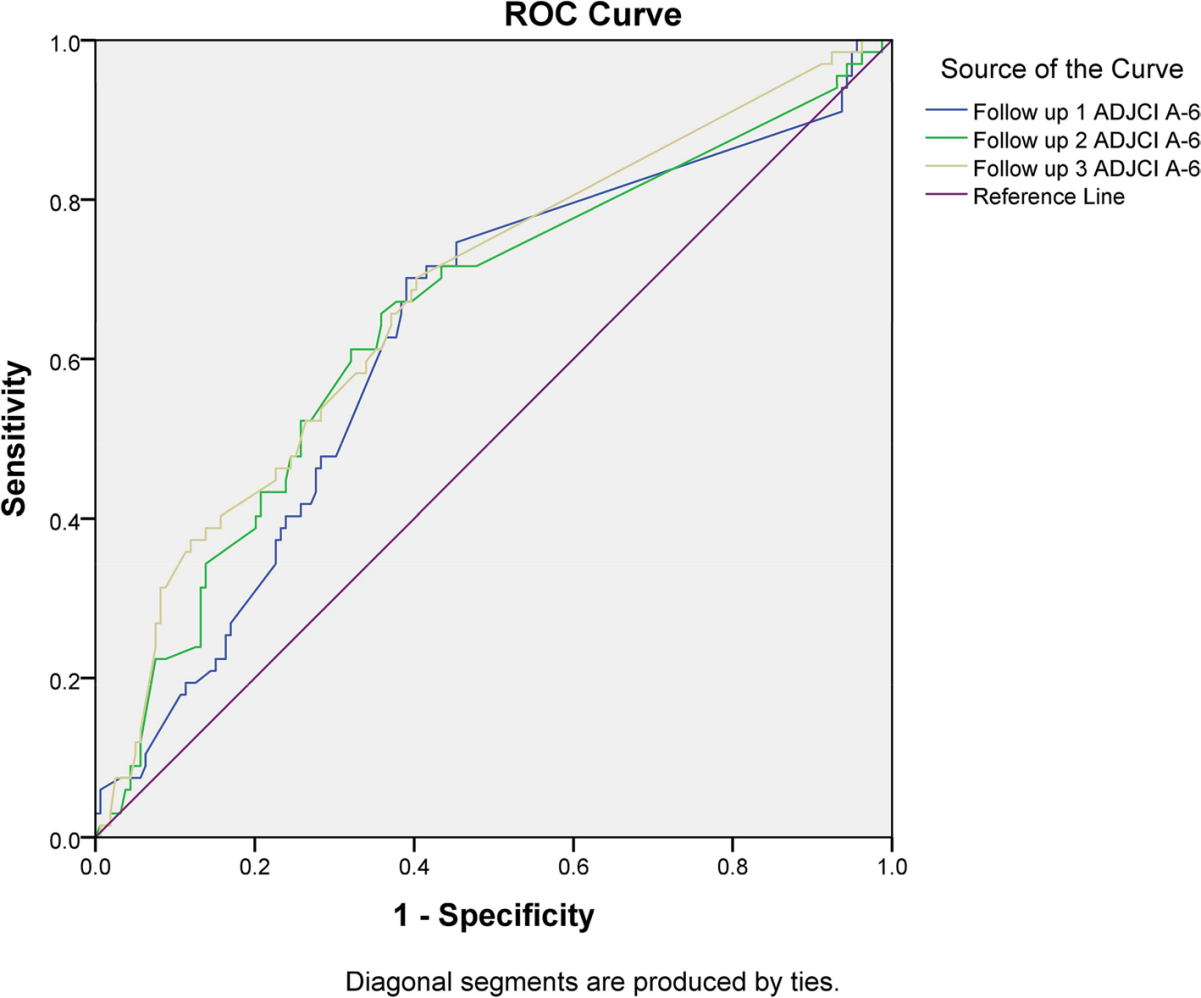
ROC at 6, 12 and 18 months

For evaluating the predictability of the reduced Cariogram, ROC curves were generated using adjusted caries increments and baseline risk levels (Fig. 1). For the reduced Cariogram model, the area under the curve in the first follow-up period was 0.63 (95% CI 0.55–0.71; *p* < 0.002). Reduced Cariograms demonstrated a sensitivity of 70% and specificity of 60% using 31.16 as the cutoff value.

For the reduced Cariogram model at 12 months, the area under the curve was 0.65 (95% CI 0.57–0.73; *p* < 0.0001). The reduced Cariogram's sensitivity was 66% and specificity was 64% when using a cutoff value of 29.82.

The reduced Cariogram model had 0.70 area under the curve (95% CI 0.59–0.74; *p* = 0.0001) and predicted caries increment at the third follow-up (18 months). On a threshold value of 29.90, the reduced Cariogram displayed a sensitivity of 70.15% and a specificity of 60%.

## Discussion

Our research aimed to evaluate the statistical significance of the reduced Cariogram in predicting caries increments across various risk levels. In this study, we implemented a reduced Cariogram model that eliminated the need for saliva and bacteria testing. Given that salivary flow abnormalities are extremely rare in children [14], salivary and microbial testing become less relevant. Furthermore, saliva and microbial testing are technique-sensitive, time-consuming, and expensive [15]. A study on 11–12-year-old Pakistani children demonstrated that a reduced Cariogram, even without saliva and microbial testing, could effectively identify children at risk for caries [16]. However, the predictability of the reduced Cariogram has yet to be studied for Pakistani populations.

We evaluated the diagnostic accuracy of the Cariogram using the area under the receiver operating curve (ROC). According to the evidence, diagnostic models with an area under the curve more than 0.50 can differentiate between individuals with or without outcome [17]. In our study, the accuracy of reduced Cariogram was 0.63 after six months, 0.65 after 12 months, and 0.70 after 18 months. Multiple studies have shown consistent results of reduced Cariogram after 12 to 24 months [5, 6]. However, barely any study has been carried out on the prediction of a reduced Cariogram after six months. Previously, 12–24 months have been reported to be required to verify Cariogram predictability statistically significant [5, 6, 18]. In this study we found that reduced Cariograms can also predict caries progression as early as six months in advance. In this study poor accuracy of reduced Cariogram after six months follow up could be due to the low caries increment.

In our study, the sensitivity and specificity of the reduced Cariogram was 66% and 64% at 12-months and 70.15% and 60% at 18-months. Similarly, a two-year prospective study on schoolchildren reported that the reduced Cariogram had high sensitivity and low specificity [5]. After 12 months, we observed low sensitivity and specificity in our study, possibly resulting from low caries increment or regression of early caries lesions.

Our results indicate that the reduced Cariogram demonstrates higher sensitivity compared to specificity. Sensitivity and specificity are crucial parameters in evaluating the performance of a diagnostic tool. Sensitivity represents the proportion of true positives (correctly identifying individuals at risk of dental caries), while specificity represents the proportion of true negatives (correctly identifying individuals not at risk). In the context of a risk assessment tool for dental caries, the emphasis on either sensitivity or specificity depends on the clinical implications and goals. Therefore, in case of Pakistan where dental caries prevalence is high, a diagnostic tool with higher sensitivity is preferable as it aids in early identification of at-risk individuals and allows for timely preventive interventions, contributing to improved population health outcomes. Similarly previous research highlighted the importance of identifying patients with a higher risk of developing dental caries over those at a lower risk [18, 19].

However, a risk assessment tool that is overly sensitive to caries may yield false-positive results, potentially leading to overtreatment of patients. The use of tests with higher sensitivity could lead to elevated preventative costs. Nevertheless, if practitioners apply clinical judgement and consider comprehensive approach to preventive care, tailored to individual patient needs, to optimize the benefits of risk assessment tools with higher sensitivity while mitigating potential drawbacks [20, 21].

In all caries risk groups, the mean caries increment increased from baseline to follow-up. Studies conducted by Sudhir et al. [18] and Peterson et al. [19] also reported a similar trend. Furthermore, we found that high-risk children had a greater mean caries increment than low-risk children at all follow-ups. Similarly, other studies also found that high-risk children experienced ten-fold more caries increments than low-risk children [22, 23]. The rise in consumption of cariogenic foods and sugars among high-risk children may be the cause of this trend. Similarly, a study conducted on Pakistani school children reported an increase in consumption of sugar and cariogenic food intake [7].

### Application of Cariogram in Pakistan

It is evident that in Pakistan, budget allocations for oral health care services are constrained due to the priority given to controlling communicable diseases with high mortality rates [24]. Furthermore, the absence of community and school-based dental programs offered by the Ministry of Health, coupled with the distribution of dental practitioners primarily in urban areas, has led to challenges in extending oral health promotion activities to a wider population [24]. Based on these limitations, the rational approach to adopt in improving the oral health and especially the caries status of its 11- 12 years-old children is to provide oral health care services at appropriate intervals on the basis of individual caries risk level. It can reduce the burden on the health care system by identifying an individual in genuine need of dental treatment.

Given these circumstances, we concur with the perspective that the Cariogram's widespread application in the community setting may indeed be intricate. We acknowledge that its potential practical unattainability at a large scale could hinder its effectiveness in real-world scenarios, potentially leading to non-effective interventions. Given these considerations, it's important to weigh the potential benefits of the Cariogram against the challenges inherent in its large-scale implementation in Pakistan. While its utility for assessing individual caries risk is well-established, addressing the aforementioned limitations becomes crucial to enhancing its effectiveness.

Raising awareness about the significance of oral health and its impact on overall well-being might encourage increased budget allocation for oral health care services, making resources available for Cariogram implementation. On the other hand, we supports the adoption of a risk-based approach for oral health care services in Pakistan, particularly for the 11–12 years-old children, who face a significant burden of caries. The risk-based approach, as evidenced by international research [25, 26], offers the advantage of optimizing resource allocation by identifying individuals genuinely in need of dental treatment. Within this context, the reduced Cariogram presents itself as a viable solution due to its suitability for low-resource settings [16, 27]. Notably, the application of the reduced Cariogram requires limited resources, is easy to comprehend and implement, and does not necessitate saliva and microbial testing.

We also acknowledge that certain aspects of the risk assessment process might require further attention. For instance, collecting dietary information through methods like 24-h dietary recall can be time-saving and practical, considering the limitations of community settings. Similarly, adapting the assessment of risk factors such as plaque measurements and access to fluoride sources can be achieved through clinical examination and questionnaires.

We recognize that computerization of dental practices is underway in Pakistan, facilitating the integration of this tool. Furthermore, introducing comprehensive training programs, especially in rural and underserved areas, can empower healthcare providers to effectively use the Cariogram in risk assessment and intervention planning.

In conclusion, while challenges related to cost, complexity, lack of trained personnel, and technology accessibility could limit the Cariogram's implementation in Pakistan, strategic adaptations and focused efforts to address these challenges can potentially enhance its practicality and impact. By tailoring its implementation to the unique needs and constraints of Pakistan's oral health care landscape, the Cariogram has the potential to play a valuable role in identifying at-risk individuals and guiding effective interventions.

The strength of this study lies in its novelty as the first longitudinal research on the predictability of the reduced Cariogram conducted in Pakistan, thus providing unique and valuable insights. It further offers data on the prevalence of dental caries among 11–12-year-old school children in Bhakkar. To prevent excessive evaluation of participants and maintain a balanced assessment of the outcomes, a teacher, nominated by the administrator, randomly selected students from the provided list.

In this study, we did not employ radiographs to evaluate caries, which may have resulted in an underestimation of both caries prevalence and the caries increment rate. Despite securing participants' contact information and sending reminders via phone or message, we observed a high dropout rate. Following the guidelines for health studies with over 5% missing data [28], we [29] performed multiple imputations to address the issue of missing data in our study.

## Conclusion

Our findings indicates that a reduced Cariogram can predict the progression of caries in both cavitated and non-cavitated lesions and model exhibits a level of discriminatory ability. While it might not achieve a very high accuracy, it suggests that the model is able to predict caries increment effectively than random guessing. Based on the results, it may be feasible to use a reduced Cariogram in Pakistan for predicting the incidence of caries and strategically distributing resources to those individuals who require them most.

## Data Availability

The data that support the findings of this study are available from the corresponding author upon reasonable request.

## Abbreviations

ICDAS: International caries detection and assessment systems
ADJCI: Adjusted caries increment
